# A web-based resource for exercise training in children treated for brain tumours to improve cognitive sequelae: Development and usability

**DOI:** 10.1177/20552076241272710

**Published:** 2024-09-05

**Authors:** Elizabeth Cox, Marium Kiwan, Cynthia de Medeiros, Jeanne Chen-Lai, Celia Cassiani, Julie Tseng, Krista Johnston, Brian W Timmons, Jennifer N Stinson, Eric Bouffet, Donald J Mabbott

**Affiliations:** 1 7979 Hospital for Sick Children, Toronto, Ontario, Canada; 2 7938 University of Toronto, Toronto, Ontario, Canada; 3 McMaster Children's Hospital, Hamilton, Ontario, Canada; 4 3710 McMaster University, Hamilton, Ontario, Canada

**Keywords:** eHealth, cognitive dysfunction, paediatric brain tumour, exercise, therapeutics

## Abstract

Improving cognitive sequelae in children treated for brain tumours (CTBT) requires accessible interventions. While instructor-led exercise in a hospital setting is efficacious, it is not extended to communities.

**Objectives:**

We aimed to (i) develop a website with educational resources/tools for community health and fitness professionals (HFP) to deliver exercise for CTBT in community settings to improve cognition and (ii) assess its usability by community HFP. It was hypothesized that the website would be learnable, clear, satisfactory and efficient to deliver exercise.

**Methods:**

A scoping review determined the state of eHealth resources to support exercise for CTBT and identified knowledge and resource gaps. Three focus groups with HFP who served cancer survivors in hospital or community settings (*n* = 13) identified user needs; content analysis identified themes. Gaps from the scoping review and themes from focus groups informed website content. A questionnaire assessed its usability by community HFP (*n* = 4). Descriptive statistics inferred the website's learnability, clarity, satisfaction and efficiency. Open-ended responses identified issues.

**Results:**

The scoping review revealed a lack of eHealth resources supporting exercise to improve cognition in CTBT and education for HFP to deliver exercise. Six themes were identified in the focus groups. HFP rated the website as sufficiently learnable, clear, satisfactory and efficient. Two minor issues were reported and addressed.

**Conclusion:**

The website marks one of the first eHealth resources to increase accessibility of intervention to improve cognitive sequelae and ultimately quality of life in CTBT. HFP also gain access to education and tools to deliver exercise in community settings.

## Introduction

The detrimental impact of cognitive sequelae in children treated for brain tumours (CTBT) has prompted a thorough characterization of their impact on functional outcomes, which has been found to be significant.^1,2,3,4,5^ The importance of developing effective and accessible interventions to improve cognitive outcomes in patients cannot be overstated. Although advances in treatment have led to increased survival rates,^6^ CTBT often experience long-term cognitive sequelae^1,2,3,7,8^ that are associated with difficulties in academic and social domains. For example, slower information processing speed in CTBT is associated with a history of repeating a grade in school, which is likely indicative of difficulty meeting academic requirements.^5^ Patients also score more poorly on measures of attention relative to normative scores, and poor attention predicts worse performance on reading and mathematical reasoning.^1^ Further, deficits in cognitive control are negatively associated with social functioning in patients, such that greater deficits are related to reduced social skills.^3^

Interventions must be developed to promote improvements in long-term cognitive sequelae following paediatric brain tumour and treatment. Physical exercise, which is defined as planned, structured and repetitive physical activity to improve health and maintain fitness, is an excellent strategy which has given its cost-effectiveness and convenience. Physical activity is any bodily movement produced by skeletal muscles that requires energy expenditure.^9^ There is now consensus that physical activity is beneficial and safe for paediatric cancer survivors^10^ and evidence that exercise improves cognition in typically developing and overweight children, and CTBT.^11,12,13,14,15,16,17^ We recently demonstrated the safety and feasibility of a 12-week clinical trial of group exercise training to improve cognitive outcomes, including information processing speed, attention and cognitive control, in CTBT following the completion of treatment (i.e. in remission); ClinicalTrials.gov: NCT01944761).^15,16,17^ The trial was led by health and fitness professionals (HFP) with expertise in paediatric neuro-oncology in a hospital setting (i.e. hospital-led). Notably, there were no adverse effects of exercise training, and adherence and retention rates were high (84% to 100%).^15^ Most patients receive their post-treatment rehabilitative care in the community, however, making hospital-led interventions challenging to access. And while community- and home-based interventions for CTBT exist, to-date they target social skills and psychosocial functioning^19,20^ or require that patients have direct access to technology, such as a computer, which can be costly and less convenient.^18,^^21,22^ Further, the coronavirus 2019 pandemic highlights the need for these interventions if and when hospital infrastructure is unavailable. It is therefore critical to develop accessible interventions in community settings, such as group physical exercise led by community HFP (i.e. community-led), to improve cognitive outcomes and promote successful functioning for the majority of patients.

By developing community-led interventions for CTBT, proof of principle could be established for viable therapeutic approaches for other forms of paediatric acquired brain injury. Despite their potential value, there are limited interventions in community settings more generally, with reported lack of sufficient rehabilitative service delivery.^23,24,25^ For example, a follow-up study of young children hospitalized for acquired brain injury showed that most patients were discharged with no referral to outpatient education or rehabilitation resources. Further, 20.7% of the sample reported having unmet educational needs 8.5 years later and service receipt declined over time.^26^ A systematic review of community- and home-based interventions for adolescents following traumatic brain injury also found there is still a lack of high quality evidence to support their more general use as community rehabilitation tools.^27^

eHealth is well-positioned to support the delivery of interventions in community settings, as it links clinical and everyday environments by providing healthcare services efficiently and cost-effectively in a variety of settings.^28^ Here eHealth is defined as a set of technologies applied through websites, smartphone and tablet applications to deliver healthcare services^29^ and support health education, knowledge and research.^30^ It is already utilized in a variety of settings related to paediatric oncology,^31,32^ including education and support for families of children and adolescents with cancer^33^ and pain assessment for adolescent cancer survivors.^34^

Given the suitability of exercise as an intervention to improve cognitive sequelae in CTBT, the need for greater access to interventions in the community, and its cost-effectiveness and convenience, we sought to develop an eHealth resource to support exercise training as intervention. The ‘Fitness to Aid the Brain and Cognitive Skills’ (Fit ABCS) website is designed to provide educational resources and tools for community HFP to deliver exercise training to CTBT in community settings with the goal of improving cognitive outcomes. The website caters to HFP in accordance with our prior trial of hospital-led group exercise training, which demonstrated an instructor-led model is safe, feasible and efficacious to improve cognitive outcomes in patients.^15,16,17,35^ The website was developed in collaboration with a network of community support centres that provide services for individuals diagnosed and treated for cancer, including exercise programming delivered by community HFP. The knowledge and expertise of these professionals was leveraged to aid in the website's co-design and development.

The Fit ABCS website is comprised of content that is organized into the following modules: (i) an Instructor Training Module for HFP to learn to work with CTBT in exercise contexts and deliver exercise training to patients in the community, (ii) an Activity Library Module comprised of 67 exercise activities that include written, photo and/or video instructions, modifications and progressions, (iii) a Session Planner Module to record programme information (e.g. instructors present, location) and select activities for each exercise session and (iv) a Session Report Module to provide tools for instructors to document relevant information following each exercise session (i.e. participant attendance, observations about the exercise sessions, safety incidents). The website also includes a Tips Sheet that is easily accessible to instructors during exercise sessions. The Tips Sheet provides a summary of the Instructor Training Module to help instructors deliver exercise training, such as how to choose appropriate activities and ensure safety. See Supplemental Materials for a video overview of the website.

The purpose of this report is to describe the development of the Fit ABCS website and determine its usability by community HFP. Specifically, to develop the website we (i) first aimed to assess the current state of eHealth resources to support exercise training as intervention for CTBT and paediatric acquired brain injury more broadly (e.g. availability, type of exercise, breadth of programming). This assessment allowed for identification of remaining knowledge and resource gaps. (ii) We then aimed to create content, and design and build an eHealth resource (i.e. the Fit ABCS website) that would address these gaps and support HFP to deliver exercise training to CTBT in the community. (iii) Once the website was built, we aimed to evaluate its usability by determining its learnability, clarity, satisfaction and efficiency to deliver exercise training to CTBT in the community. It was hypothesized that the website would be rated as sufficiently learnable, clear, satisfactory and efficient to use to deliver exercise training to CTBT in the community.

## Methods

**Figure 1.**
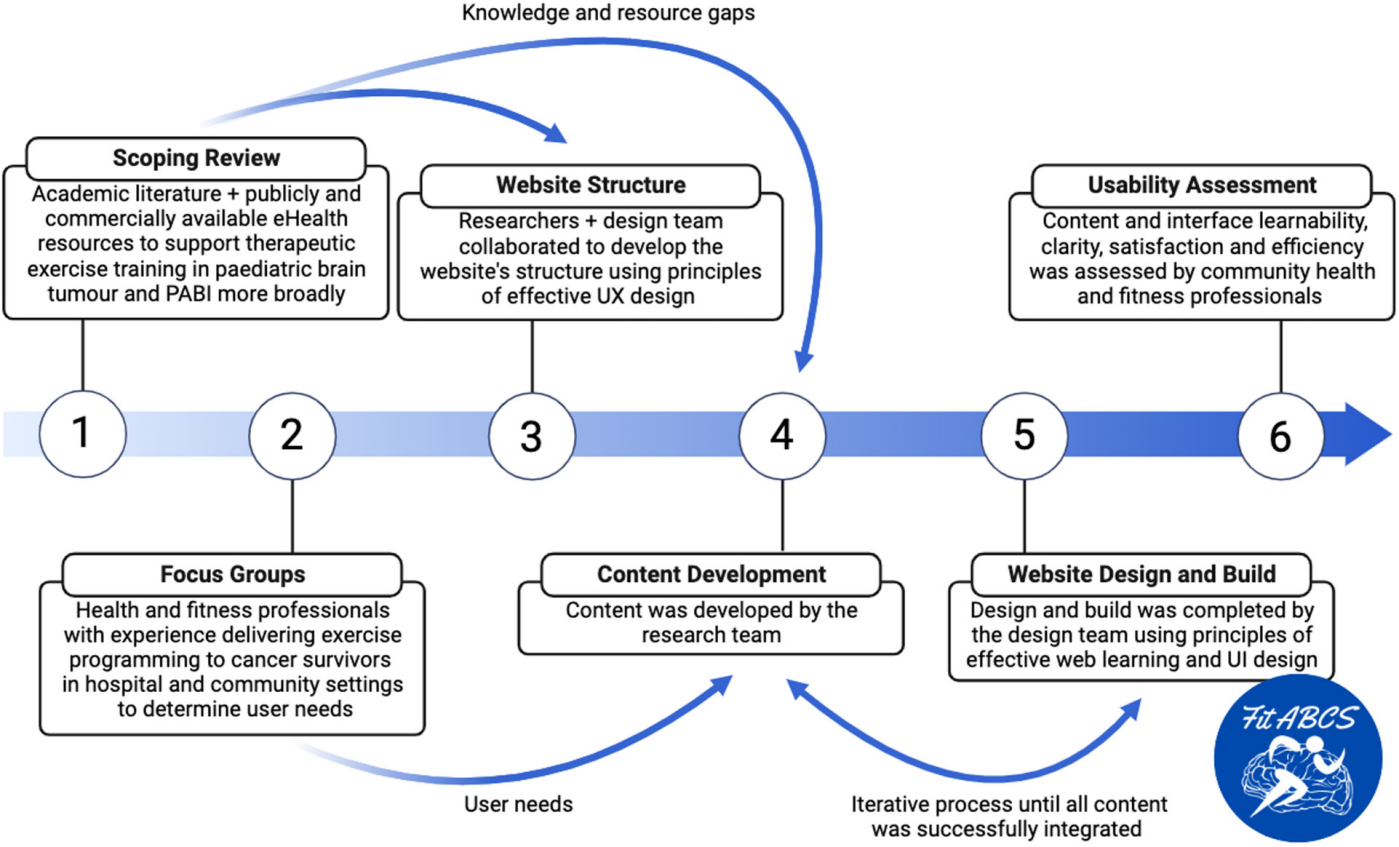
Process of development and usability testing of the Fit ABCS website. Designers denote experts in paediatric healthcare education, and user experience and interface design at the hospital for sick children.

PABI: paediatric acquired brain injury; UX: user experience; UI: user interface.

A scoping review was first conducted to investigate the current state of academic literature, and publicly and commercially available eHealth resources to support exercise training as intervention for children and adolescents who experienced an acquired brain injury – with a particular focus on those treated for a paediatric brain tumour. Focus groups were then conducted to determine user needs for the Fit ABCS website. HFP with expertise delivering exercise programming to cancer survivors in hospital and community settings took part, as the Fit ABCS website uses an instructor-led model to deliver exercise training. Website content development was informed by an understanding of remaining knowledge and resource gaps pertaining to eHealth resources to support exercise training and information derived from the focus groups. Its interface was then designed and built. Lastly, usability testing was conducted with an additional set of community HFP to assess the website's learnability, clarity, satisfaction and efficiency. See Figure 1 for a visual representation of the methodological process. Informed consent was not obtained by HFP. As discussed in the Introduction (page 4), these professionals were considered collaborators who provided knowledge and expertise to aid in the website's co-design and development. The Fit ABCS website was developed to support the delivery of an ongoing clinical trial of exercise training for CTBT in community settings (see ClinicalTrials.gov: NCT05367076 and Discussion, Comparison with Prior Work and Future Directions, page 29). CTBT are therefore considered participants in this research and their informed consent (or assent) is obtained prior to their participation in the clinical trial.

### Scoping review

To assess available eHealth resources that support the delivery of exercise interventions to CTBT, searches were completed from September to December 2019 and October 2022. The search strategies are described below. The scoping review was completed by one reviewer (EC).

#### Academic literature

A scoping review was performed using methods specified in the Preferred Reporting Items for Systematic reviews and Meta-Analyses extension for Scoping Reviews;^36,37^ a review protocol specific to this study is not available. The electronic databases PubMed and PsyINFO were searched. Publication date limits were not applied. Search terms were used to identify published journal articles related to exercise, acquired brain injury, brain tumour, cancer survivorship and eHealth (i.e. websites, and smartphone and tablet applications; see Supplemental Table 1 for search terms). The complete PsyINFO search strategy is shown in Supplemental Table 1 and is analogous to the other database search performed.

Titles and abstracts were retrieved for identified records. Duplicates and retracted articles were removed and the titles and abstracts of remaining articles were screened using the following criteria: (i) exercise intervention, (ii) delivered with the support of an eHealth resource or electronic device (e.g. Wii Fit, DVD, telephone), (iii) to individuals treated for cancer or acquired brain injury and (iv) published in the English language. Electronic devices were included to capture interventions that primarily used these devices with support from eHealth resources (e.g. see Sable et al., 2017). The scoping review focused on primary research, which reports on original study findings, including design, methods and interpretation. To maintain focus on primary research, letters, case studies, meta-analyses and reviews were excluded. Meta-analyses and reviews introduce redundancy because they synthesize evidence from primary research. Although case studies offer insights into individual experiences, they lack systematic evaluations of interventions. Letters, while serving as platforms for commentary or new viewpoints on existing research, do not provide comprehensive reporting of original study findings. Prior to excluding meta-analyses and reviews, the snowball method was applied to identify other relevant articles, whereby references in one article were the source for finding other relevant articles. Full-text articles that met the screening criteria were subjected to the following eligibility criteria: (i) the study aimed to evaluate the feasibility or efficacy of the exercise intervention, (ii) the intervention was delivered with the support of an eHealth resource, (iii) to children and adolescents aged 6–17 at the time of intervention, (iv) treated for a brain tumour or acquired brain injury more broadly. Full-text articles were also eligible if they (v) aimed to evaluate the experience of instructors delivering the exercise intervention using an eHealth resource. Research reporting on interventions in CTBT ages 6–17 were included because patients who participated in the hospital-led clinical trial were also within this age range. Given that the Fit ABCS website content was modelled on content from the hospital-led trial, the scoping review that informed the website's development also aimed to determine the state of the literature within this age range. Data from eligible full-text articles were charted using an electronic form developed by the researcher. For variables included in the data charting form and their definitions, see Table 1.

**Table 1.** Data charting form for (A) academic literature, and (B) publicly and commercially available eHealth resources that met the scoping review eligibility criteria.

Title; Developer/Author	eHealth modality	Population/End users	Exercise modality	Aim	Summary of features; Contributors	Accessibility
A) Effects of physically active video gaming on cognition and activities of daily living in childhood brain tumor survivors: a randomized pilot study; Sabel et al., 2017^a^	Web-based	CTBT 7–17 years of age	Multimodal	Improve cognition and the execution of activities of daily living	Features: 10–12 weeks of active video gaming using a motion-controlled video console and web-based coaching sessions to sustain motivation and evaluate enjoyment; Contributors: developed by research scientists and clinicians. End users were not involved in development and/or co-design	Available through enrolment in a clinical trial
Active video gaming improves body coordination in survivors of childhood brain tumours; Sabel et al., 2016^a^	NA	NA	NA	Improve physical functioning and determine energy expenditure during active video gaming	NA	NA
A Pilot Study Evaluation of a Web-Based Token Economy to Increase Adherence with a Community-Based Exercise Intervention in Child and Adolescent Cancer Survivors; Gilliam et al., 2011	Web-based	Paediatric cancer survivors 6–18 years of age	Multimodal	Increase adherence to an exercise intervention using a web-based token economy and improve aerobic, and improve aerobic fitness and physical functioning	Features: 6 weeks of exercise training, web-based exercise logs and token economy (participants earned ‘chips’ for adherence to the intervention); Contributors: developed by research scientists and clinicians. End users were not involved in development and/or co-design	Available through enrolment in a clinical trial
B) Cancer and Exercise Certification for Health and Fitness Professionals; Thrive Health Services	Web-based	HFP	Multimodal	Training programme and certification for HFP to become experts in evidence-based exercise planning and support for cancer survivors	Features: Video-based online training modules and tools, printable handbook, certification upon completion; includes paediatric cancer and exercise module; Contributors: developed by research scientists and clinicians. End users were involved in development.	Commercially available; Paediatric cancer and exercise module available at additional cost
Pediatric Oncology Exercise Manual (POEM); Health and Wellness Lab, Faculty of Kinesiology, University of Calgary; 2014^b^	Web-based (printable)	HFP, Educators, Clinicians, Caregivers	Multimodal	Increase awareness of the benefits of physical activity for patient families and provide professionals with evidence-based information and tools to support physical activity in patients; Provider and Caregiver versions available	Features: Information on paediatric oncology, subtypes of paediatric cancer and physical activity recommendations; includes discussion on active video gaming; Contributors: developed by research scientists and clinicians. End users were involved in development.	Publicly available
Yoga Thrive Youth; Long et al., 2015	Web-based (printable)	Paediatric cancer survivors, Caregivers	Yoga	Improve patient health and wellness by increasing physical fitness, self-esteem and relaxation	Features: Information on the use of yoga in paediatric oncology, breath, yoga and relaxation practices; Contributors: developed by research scientists and clinicians. End users were involved in development and co-design.	Publicly available

*Note*: eHealth modalities are defined as web, smartphone or tablet-based applications; Population/End users are defined as individuals who are intended to use the eHealth platform; Exercise modality is defined as the type of exercise performed and included cardiovascular, strength, flexibility, balance, coordination and mobility; Aim is defined as the purpose of the platform; Summary of features denotes the key characteristics of the eHealth platform and contributors denotes the types of professionals who aided in the platform's development; Accessibility is defined as platform's mode of availability. Commercially available resources are for purchase. Publicly available resources and those accessible through a clinical trial are free of charge.; NA denotes information that is shared between records.

Abbreviations: NA: not applicable; HFP: health and fitness professionals; CTBT: children treated for brain tumours.

^a^ Records report on separate outcomes from the same clinical trial.

^b^ Encompasses printable materials used in the Thrive Health Services cancer and exercise certification for health and professionals.

#### Public and commercial eHealth resources

The Apple App Store, Google Play Store and Google Search Engine were used to search for publicly and commercially available eHealth resources related to exercise, acquired brain injury, brain tumour and cancer survivorship by one reviewer (EC). Search terms included (physical activity or exercise) and (acquired brain injury or cancer or brain tumour). Terms from each group were submitted in an iterative fashion until all combinations of terms were applied. Names and product descriptions were used to screen for relevant eHealth resources using the following criteria: (i) exercise intervention, (ii) delivered to individuals treated for cancer or acquired brain injury, (iii) published in the English language. eHealth resources that met the screening criteria, were then subjected to the following eligibility criteria: (i) the resources aimed to support delivery of exercise training, (ii) to children and adolescents aged 6–17, (iii) treated for a brain tumour or acquired brain injury more broadly. eHealth resources also met eligibility criteria if they (iv) aimed to educate exercise professionals on how to administer exercise training. Data from eligible publicly and commercially available eHealth resources were charted using the same electronic form as that used for academic literature (see Table 1 for included variables and their definitions).

The charted data were synthesized to determine the current state of eHealth exercise interventions for CTBT and paediatric acquired brain injury more broadly and identify knowledge and resource gaps. To synthesize the data, the number of eHealth resources that met eligibility criteria was reported and key characteristics were summarized, including the eHealth mode of delivery, population/end users, programme structure and accessibility.

### Website development

#### Focus groups

To determine user needs for the Fit ABCS website, three focus groups were conducted with four to five HFP per group (*n*_*total*_= 13). Smaller group size increases the likelihood that members will demonstrate adequate participation and interaction with one another. Moderators can also more easily manage and attend to smaller groups.^38^ One focus group was conducted with registered physical therapists with specialized education and training in paediatric neuro-oncology who had delivered the 12-week hospital-led trial of exercise training to CTBT (*n* = 5). Two focus groups were conducted with kinesiologists and exercise physiologists who had experience delivering exercise programming to children and adults diagnosed and treated for cancer at the network of community support centres (*n* = 8, 4 per focus group). These HFP completed an online course on how to lead the support centre's exercise programme and work with adult cancer survivors.

Each focus group was moderated by three research team members (CdM, CC and EC) using semi-structured interview guides informed by guides used in previous work to assess the usability and feasibility of eHealth resources for paediatric clinical populations, including oncology.^34,39^ Interview topics included instructor education on how to work with CTBT in the exercise contexts, activities to perform during exercise sessions, and information capture, such as attendance and safety incidents. Each interview was two hours in length and no other individuals besides the moderators and professionals were present. Two members of the research team (JLC and EC) transcribed interview audio-recordings verbatim. The moderators read the data to collectively identify data codes and then independently coded the data using a line-by-line technique based on the objective of identifying user needs for the website. Content analysis was used to group codes into categories based on between-code relationships. Categories were then grouped into themes (Supplemental Table 2).^40,41,42^ Inter-rater reliability was high, as there were no differences in opinion regarding categories and themes.

#### Content development and website design

The content was developed by members of the research team and informed by knowledge and resource gaps identified through the scoping review, qualitative themes derived from the focus groups, consultations with members of the research team with expertise in physical therapy, psychology and paediatric neuro-oncology, consultations with a volunteer committee of patients, family members, researchers, and clinicians at the Hospital for Sick Children, and a paper-based manual. The manual was previously used by HFP to deliver the 12-week hospital-led trial of exercise training for CTBT. Given the hospital-led trial's safety, feasibility and efficacy,^15,16,17,35^ relevant content was carried over to the website. This content included the structure of exercise training sessions, exercise activities included in the Activity Library Module and session reporting tools, such as the safety incident report included in the Session Report Module. See Results, Content Development for details regarding the development of content informed by knowledge and resource gaps identified in the scoping review, qualitative themes derived from the focus groups and consultations with experts.

The Fit ABCS website was built by a team of experts in paediatric healthcare education, and user experience and interface design at the Hospital for Sick Children (i.e. design team). The researchers first collaborated with the design team to create a website structure that achieved its goal to provide educational resources and tools for community HFP to deliver exercise training to patients in community settings while maintaining principles of effective user experience design. Once the structure was established (i.e. the website Modules), the website was built through an iterative process, whereby content was provided by the researchers, and reviewed and implemented by the design team to meet principles of effective web learning and interface design. Content that could not conform to these principles was modified by the researchers and resubmitted to the design team. The website was complete when all content was successfully implemented.

### Usability testing

Usability testing was conducted to determine the ease of use and intuitiveness of the Fit ABCS website content and user interface. The assessment was conducted with a set of HFP with expertise in delivering exercise programming to cancer survivors at the network of community support centres who had not participated in the focus groups (i.e. users; *n* = 4). Usability testing has been well-studied in the implementation of clinical information systems, including eHealth, and has been shown to be critical to their success.^43,44^ A sample size of four is sufficient to assess usability, as Nielsen's law of diminishing returns suggests that 80% of usability problems can be identified with four or five participants.^39,45^

Questionnaires are the most used method to evaluate usability of eHealth resources.^44^ Users completed a questionnaire adapted from the Telehealth Usability Questionnaire (TUQ)^46^ and the Service User Technology Acceptability Questionnaire (SUTAQ).^47,48,49^ The TUQ is used to evaluate the usability of telehealth implementation and services conducted over computer-based systems by assessing learnability, effectiveness and satisfaction. The SUTAQ aims to assess the satisfaction of eHealth resources. The questionnaire was delivered using the Research Electronic Data Capture (REDCap) system, a web-based application developed to collect data, and create databases and projects within a secure environment.^50^ Multiple choice, Likert-type and open-ended questions were used to understand the learnability, clarity, satisfaction and efficiency of the: (i) Instructor Training, (ii) Activity Library and (iii) Session Planner Modules. Learnability was defined as users’ ability to build their knowledge on the Fit ABCS website content, including the quality of information provided and users’ confidence in the knowledge they attained. Clarity was defined as the coherence of information presented, and language and visual tools used. Satisfaction denoted users’ contentment and comfort with the website content, while efficiency denoted ease of use, including the order that information was presented and ability to navigate the website. The usability of the Session Report Module was not assessed. These tools were delivered using the REDCap system to collect safety and feasibility data about the clinical trial of exercise training in community settings that the website currently supports.

HFP were provided to access the Fit ABCS website and the questionnaire. They were instructed to review each website module, navigate through the standard features and answer the corresponding set of questions before proceeding to the next module. To increase accessibility, HFP were invited to complete the questionnaire over a two-week period, whereby their progress could be saved until the questionnaire was completed or the survey period closed.

### Statistical analysis

Descriptive statistics were calculated to infer which elements of the website prototype were sufficiently learnable, clear, satisfactory and efficient. R statistical software (version 1.0.143) was used to calculate the mode of each multiple-choice and Likert-type response to determine the value that appeared most often.^51^ Responses to open-ended questions were used to identify aspects that had issues and required modification. If major usability issues were identified, iterative cycles of changes and re-testing were conducted. Major usability issues included the need for a new module or more than one issues in a given module. Minor usability issues included one issue in a given module and if identified, were changed without re-testing.

## Results

### Scoping review

**Figure 2.**
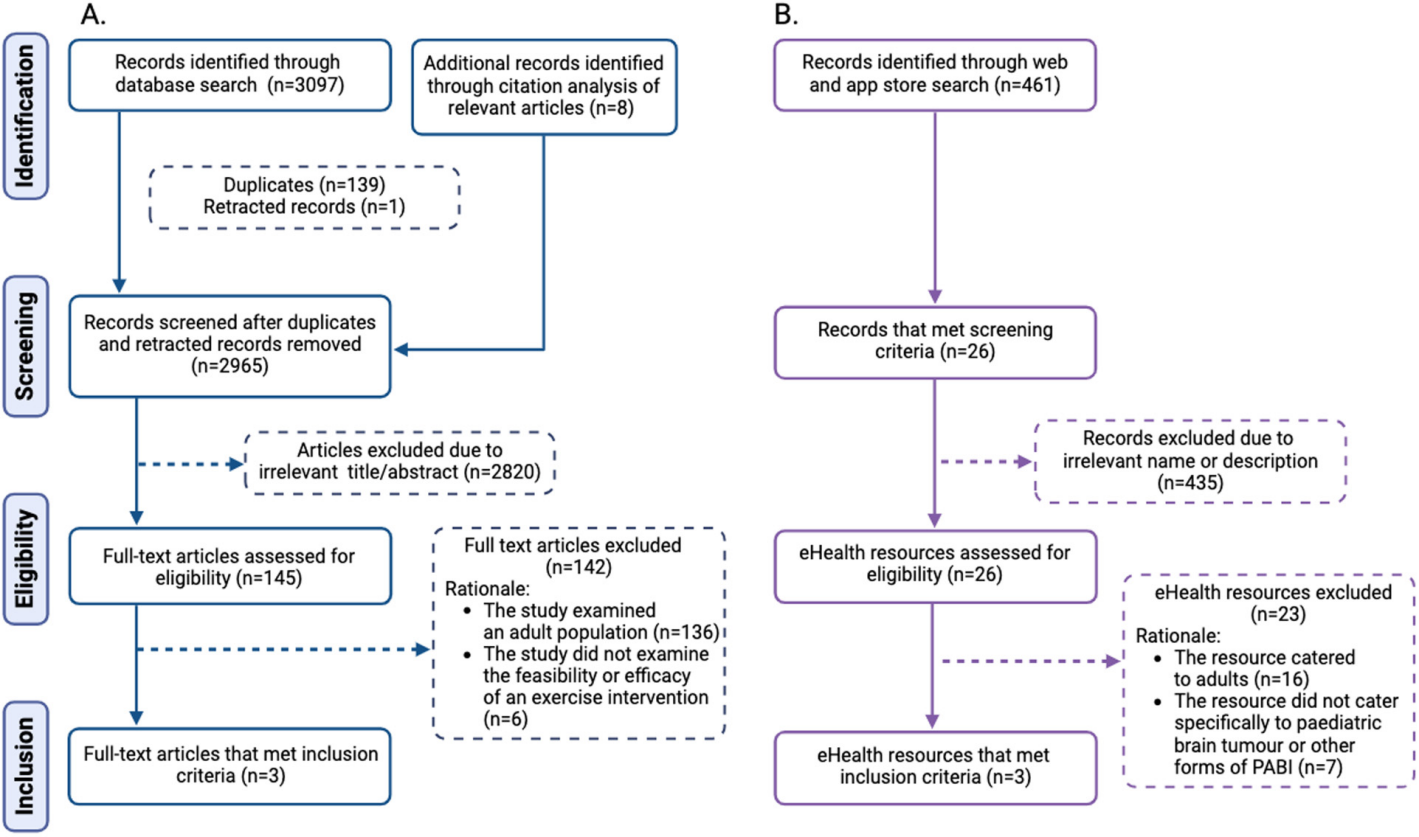
Flowchart of search process for (A) academic literature and (B) publicly and commercially available eHealth resources to support exercise training in children treated for brain tumours and paediatric acquired brain injury more broadly.

Figure 2 provides a flowchart of the process of searching for academic literature, and publicly and commercially available eHealth resources. For academic literature, records identified through PubMed were published between 1984 and 2022 and those identified through PsyINFO were published between 1982 and 2022. For public and commercial eHealth resources, records identified on the Apple App Store were published between 2007 and 2022, those on the Google Play Store were published between 2012 and 2023 and those from Google Search Engine were published between 2004 and 2022. Five eHealth resources met the eligibility criteria and were synthesized. The first two eHealth resources were clinical trials that involved active video gaming and web-based coaching to improve cognitive, motor and functional outcomes in CTBT,^18,52^ and a web-based token economy to increase adherence to an exercise intervention to improve aerobic fitness and physical functioning in paediatric cancer survivors.^53^ The third eHealth resource was a commercially available training and certification programme for HFP to deliver exercise training to cancer survivors, including CTBT.^54^ The last two were publicly available; an electronic manual that aims to increase awareness of the benefits of physical activity for caregivers/families of paediatric cancer survivors and provides professionals (i.e. HFP, educators and physicians) with evidence-based information and tools to support physical activity in these patients,^55^ and an electronic yoga manual to be used by paediatric cancer survivors and their caregivers/families to improve patient health and wellness by increasing physical fitness, self-esteem and relaxation.^56^ Both manuals discuss paediatric brain tumours and the benefits of physical activity on cognition in survivors. The eHealth resources are described in Table 1. All five were developed by research scientists and clinicians, and utilized multiple exercise modalities, while their aims features, and accessibility differed.

PABI: paediatric acquired brain injury.

The scoping review also demonstrated knowledge and resource gaps in the current use of eHealth to support exercise training for CTBT. Only five resources were identified indicating a lack of eHealth resources to support the delivery of exercise training as therapy for patients. Further, given that only two resources provide dedicated training for HFP, there is a lack of instructor-led models to deliver exercise training with the support of eHealth.

### Website development

#### Focus groups

All HFP took part in the entirety of their respective interview with no issues. Supplemental Table 2 provides the themes and categories, and example data codes from the focus groups. Interview data from professionals who had previously delivered exercise training to patients in a hospital setting were categorized into three themes: (i) education on the exercise intervention, (ii) exercise session planning and (iii) exercise session feedback. Categories that comprised the theme of education on the exercise intervention included education on CTBT in the context of exercise, the goals and objectives of the exercise training and exercise session monitoring and considerations. Exercise session planning included considerations and challenges when selecting activities, tools to aid in session preparation and instruction, and participant information. Exercise session feedback was comprised of information to collect and questions to ask about the exercise sessions.

Data from professionals with expertise delivering exercise programming to cancer survivors in the community were categorized into five themes: (i) instructor experience, (ii) instructor education, (iii) education on the exercise intervention, (iv) venue considerations and suitability and (v) exercise session planning. Categories that comprised the instructor experience theme included instructors’ prior experience working with children and adolescents, including CTBT, and credentials. Instructor education included knowledge and tools for effective programme instruction, motivation for instructors and working with children. Education on the exercise intervention included the same categories and example codes as that of professionals in a hospital setting with the addition of parent/guardian support. Venue considerations and suitability included how to incorporate exercise programme goals and objectives, and venue selection. Exercise session planning included participant information, venue information and efficiency.

#### Content development

Knowledge and resource gaps identified through the scoping review highlighted the need for (i) eHealth resources to support exercise training to improve cognitive outcomes in CTBT and (ii) instructor-led models to deliver this training with the support of eHealth. Accordingly, the authors aimed to develop content for the Fit ABCS website that would support instructors to successfully deliver exercise training to patients to promote cognitive improvement.

The themes derived from the focus groups further informed the content for the website Modules. Themes identified across focus groups were prioritized, including education on the exercise intervention and exercise session planning. For example, all focus groups expressed the need to learn about CTBT, including common physical and cognitive difficulties that impact participation in exercise. Focus group member 1 stated a need to understand the level of physical independence experienced by CTBT who participate in exercise training:

*We have clients who are not 100% physically independent. Would any of these children be with help aids? Are they all safe of independent participation, I guess is what I would want to know too.*

**Figure 3.**
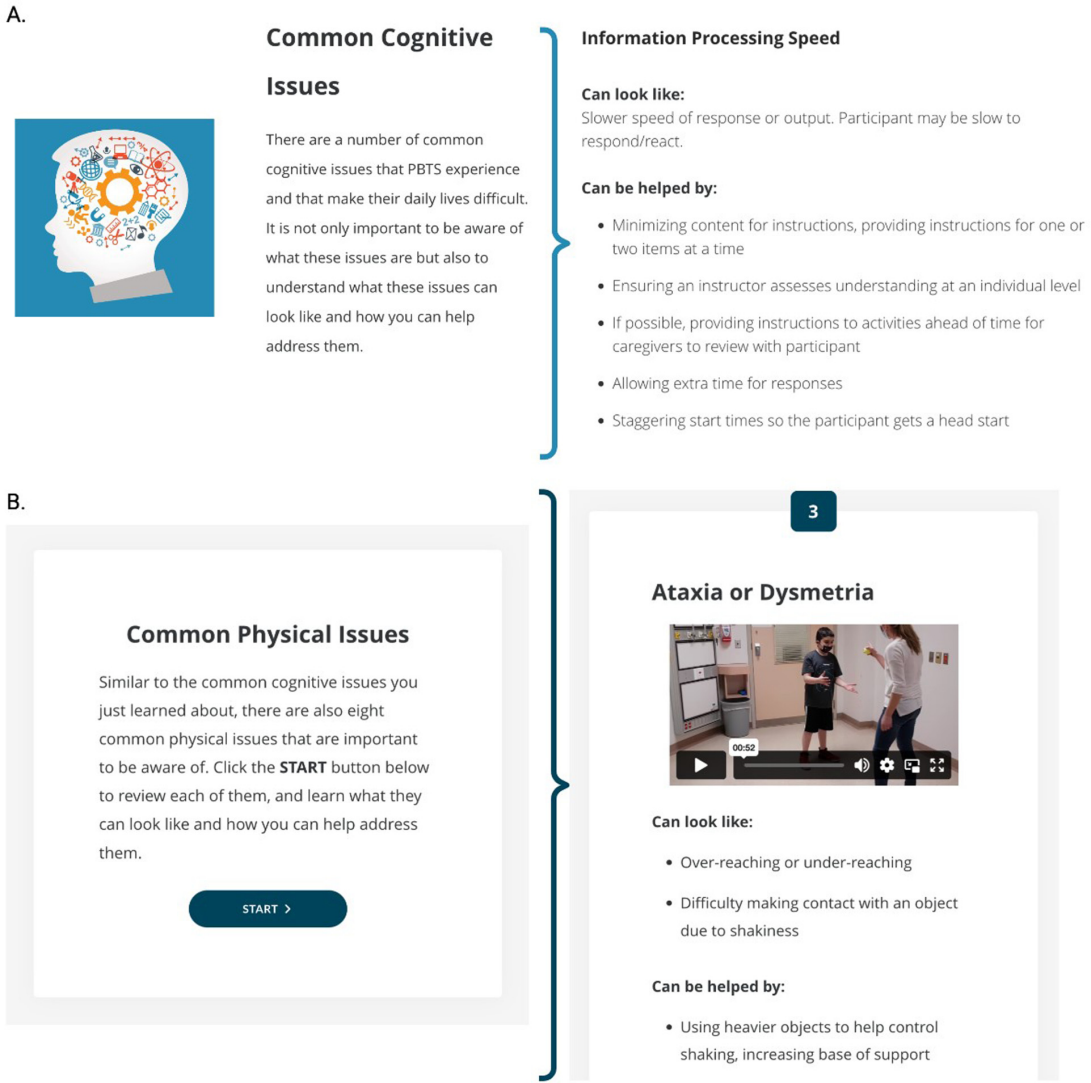
Content included in the Instructor Training Module. Descriptions and examples of common (A) cognitive and (B) physical issues experienced by children treated for brain tumours that impact participation in exercise training are shown. Suggestions to alleviate issues during exercise training are also discussed. The video includes a patient and physical therapist with expertise in paediatric neuro-oncology at the Hospital for Sick Children.

To address this need, members of the research team who are experts in physical therapy (KJ), psychology (DJM) and paediatric neuro-oncology (EB) were consulted to develop written descriptions and videos of cognitive and physical difficulties that impact patients’ participation (e.g. slower information processing speed, reduced balance and coordination); they are included in the Instructor Training Module (Figure 3). Within the theme of exercise session planning, professionals expressed a need for participant, venue and activity information. For example, focus group member 2 stated a need for comprehensive information about activities to aid session planning:

*[You should] understand what activity you want to do, how long you’re going to do it, a backup plan in case it doesn’t fly, [and] modifications that you could make to that activity depending on if a child is challenged by it or it looks too easy.*

PBTS: paediatric brain tumour survivors.

**Figure 4.**
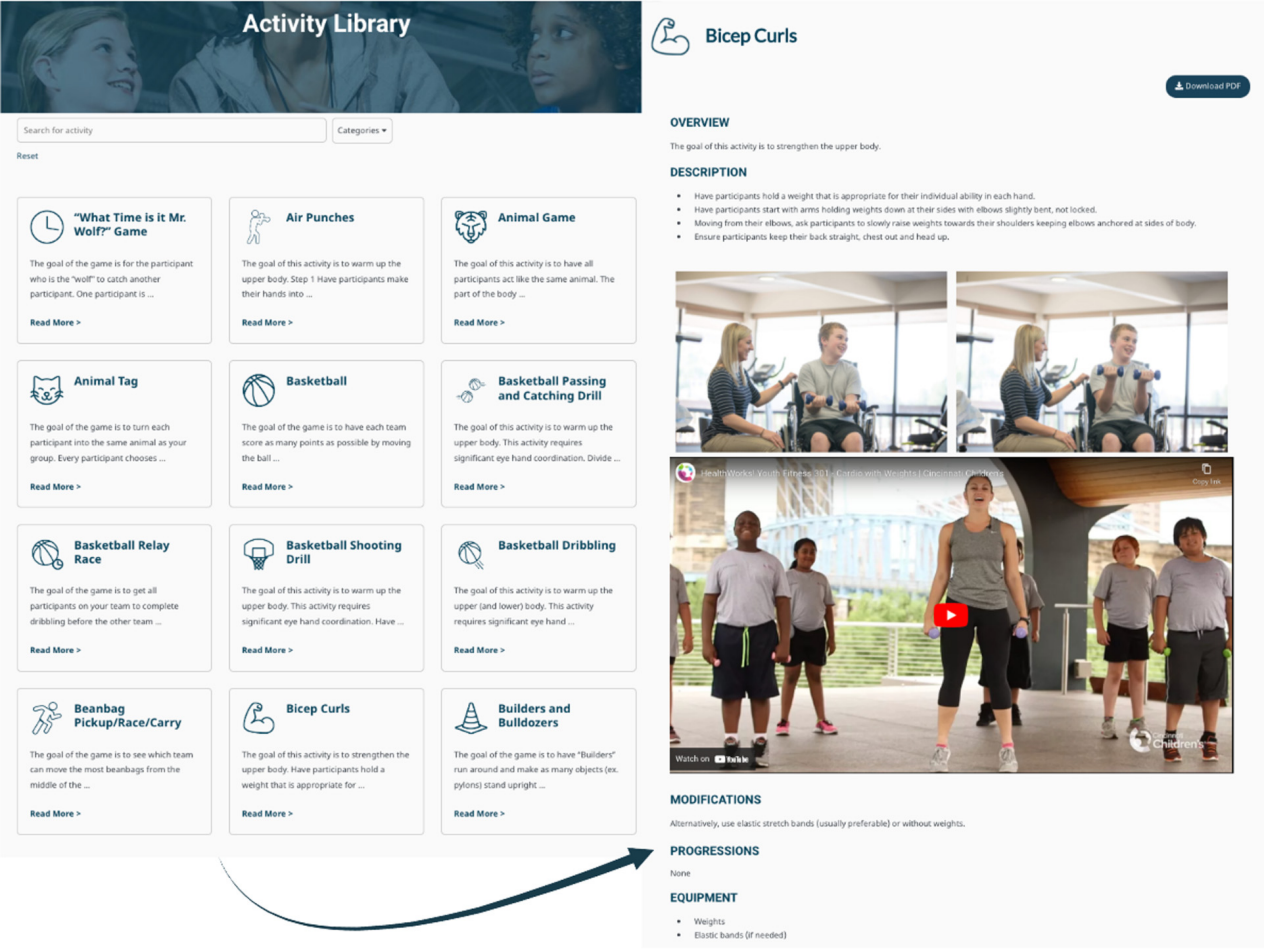
Content included in the Activity Library Module. The Activity Library includes 67 exercise activities for instructors to select from to plan exercise sessions (left). Each activity (example right) includes written, photo and video descriptions, modifications, progressions and equipment.

Accordingly, written descriptions of activities, their modifications and progressions, and visual tools (i.e. diagrams, videos and photos; Figure 4) were developed for each activity in the Activity Library Module to support successful exercise session planning. The Activity Library Module also includes a filtering system that allows instructors to easily (de)select activities based on participant needs and venue considerations (see Supplemental Video). Content was also informed by themes present only in individual focus groups, such as instructor session planning and exercise session feedback cited by professionals in a community and hospital setting, respectively.

**Figure 5.**
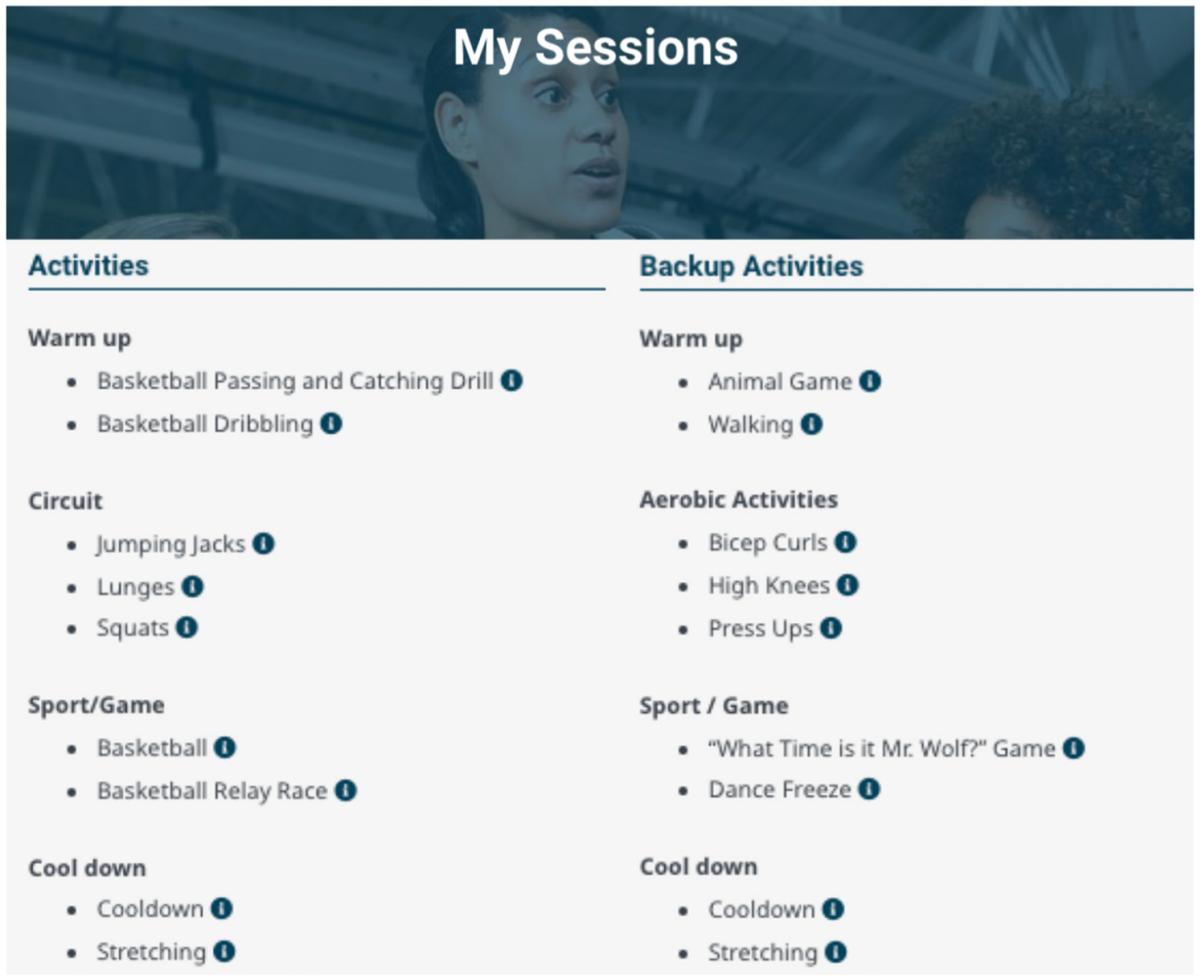
Content included in the Session Planner Module. Prior to exercise sessions instructors create session plans that include the selection of primary and alternative (‘backup’) activities.

To accommodate the need for efficient planning, the Session Planner Module allows instructors to choose alternative activities for exercise sessions to ensure participant engagement (Figure 5, see Supplemental Video). To meet the need to provide feedback, the Session Report Module prompts instructors to provide observations. By reviewing observations from previous sessions, instructors can build an understanding of (un)successful elements to optimize future sessions (see Supplemental Video). Indeed, focus group member 3 expressed a need to review observations from prior exercise sessions to inform future session planning:

*[You can see] who participated best in what, who liked [what] - there will probably be kids who liked the individual stuff way more than the game stuff - so that instructors can quickly see who participated a lot.*

Here, ‘individual’ and ‘game stuff’ refer to various components (i.e. circuit and sport/games) of each exercise session.

The volunteer committee of patients, family members, researchers and clinicians at the Hospital for Sick Children found the website's content to be sufficient with no additional feedback for improvement.

### Website usability

All users successfully completed the Fit ABCS website usability questionnaire. Users’ prior experience working with children and adolescents, including CTBT, is reported, along with ratings of learnability, clarity, satisfaction and efficiency of the website Modules (i.e. Instructor Training, Activity Library and Session Planner Modules). Usability results are displayed in Table 2. Minor usability issues reported are also described.

**Table 2.** Fit ABCS website usability questionnaire and responses by community health and fitness professionals experience working with cancer survivors (*n* = 4).

	Response	Frequency	Mode
INSTRUCTOR TRAINING MODULE			
LEARNABILITY			
How well the instructor training module provided information on the Fit ABCS programme	Excellent	4 (100%)	Excellent
	Good		
	Fair		
	Poor		
	Very Poor		
How well the instructor training module provided information on the ability to identify appropriate activities	Excellent	1 (25%)	Good
	Good	3 (75%)	
	Fair		
	Poor		
	Very Poor		
How well the instructor training module provided information on physical issues in CTBT	Excellent	3 (75%)	Excellent
	Good	1 (75%)	
	Fair		
	Poor		
	Very Poor		
How well the instructor training module provided information on cognitive issues in CTBT	Excellent	3 (75%)	Excellent
	Good	1(25%)	
	Fair		
	Poor		
	Very Poor		
How well the instructor training module provided information on the role of an instructor	Excellent	2 (50%)	Good-Excellent
	Good	2 (50%)	
	Fair		
	Poor		
	Very Poor		
Your confidence in your ability to work with CTBT after completing the instructor training module	Very Confidence		Confident
	Confident	4 (100%)	
	Neutral		
	Somewhat Confident		
	Not Confident At All		
CLARITY			
The quality of the language used in the instructor training module	Very Clear	4 (100%)	Very Clear
	Clear		
	Fair		
	Poor		
	Very Poor		
EFFICIENCY			
How easy it was to learn the content of the instructor training module	Very Easy	1 (25%)	Easy
	Easy	3 (75%)	
	Fair		
	Hard		
	Very Hard		
The ability to navigate the instructor training module (i.e. Did all the buttons work? Could you move between pages?)	Very Easy	2 (50%)	Easy-Very Easy
	Easy	2 (50%)	
	Fair		
	Hard		
	Very Hard		
The flow/organization of the content within the instructor training module (i.e. Does the order of the information make sense?)	Excellent	3 (75%)	Excellent
	Good	1 (25%)	
	Fair		
	Poor		
	Very Poor		
SATISFACTION			
Is the instructor training module missing key information that would be needed for you to successfully carry out the Fit ABCS programme?	No	3 (75%)	No
	Yes	1 (25%)	
Are there any additions to the instructor training module that you would want (i.e. would like to see) as an instructor?	No	3 (75%)	No
	Yes	1 (25%)	
ACTIVITY LIBRARY MODULE			
CLARITY			
The quality of the visual tools (i.e. diagrams, pictures and videos) used in the activity pages	Very Clear	2 (50%)	Clear-Very Clear
	Clear	2 (50%)	
	Fair		
	Poor		
	Very Poor		
Are there missing visual tools (i.e. diagrams, pictures and videos) that you feel are necessary for your understanding of activities?	No	1 (25%)	Yes
	Yes	3 (75%)	
Is there a specific form of visual tool (i.e. diagrams, pictures and videos) that you feel was missing?	No	3 (75%)	No
	Yes	1 (25%)	
The quality of the language used in the library of activities	Very Clear	2 (50%)	Clear-Very Clear
	Clear	2 (50%)	
	Fair		
	Poor		
	Very Poor		
EFFICIENCY			
The ability to navigate the activity library module (i.e. Did all the buttons work? Could you move between pages?)	Very Easy	3 (75%)	Very Easy
	Easy	1 (50%)	
	Fair		
	Hard		
	Very Hard		
How well the library of activities filters worked to identify appropriate activities	Excellent	3 (75%)	Excellent
	Good		
	Fair	1 (25%)	
	Poor		
	Very Poor		
SATISFACTION			
Your satisfaction with the activity pages and descriptions	Completely Satisfied	1 (25%)	Very Satisfied-Completely Satisfied
	Very Satisfied	3 (75%)	
	Moderately Satisfied		
	Slightly Satisfied		
	Not At All Satisfied		
To successfully use the library of activities, is there any key information that is missing?	No	4 (100%)	No
	Yes		
Are there any additions you want (i.e. would like to see) in the library of activities?	No	3 (75%)	No
	Yes	1 (25%)	
SESSION PLANNER MODULE			
EFFICIENCY			
The ability to navigate the session planner (i.e. Did all the buttons work? Could you move between pages?)	Very Easy	2 (50%)	Very Easy
	Easy	1 (25%)	
	Fair	1 (25%)	
	Hard		
	Very Hard		
SATISFACTION			
When testing the session planner, you encountered activities based on exercise category. Was there a sufficient number of activities?	Very Sufficient	3 (75%)	Very Sufficient
	Sufficient	1 (25%)	
	Neutral		
	Insufficient		
	Not At All Sufficient		
Your satisfaction with the variety of activities available to select	Completely Satisfied	2 (50%)	Completely Satisfied-Very Satisfied
	Very Satisfied	2 (50%)	
	Moderately Satisfied		
	Slightly Satisfied		
	Not At All Satisfied		
Your level of comfort using the session planner	Very Comfortable	2 (50%)	Very Comfortable-Comfortable
	Comfortable	2 (50%)	
	Neutral		
	Slightly		
	Uncomfortable		
	Not At All		
	Comfortable		
To successfully use the session planner, is there any key information that is missing?	No	4 (100%)	No
	Yes		
Are there any additions you want (i.e. would like to see) in the session planner?	No	3 (75%)	No
	Yes	1 (25%)	

#### Prior experience

All users had prior experience working with children and adolescents in camp (*n *= 2), community (*n* = 2) and private healthcare (*n* = 3) settings. Time since users last worked with children and adolescents ranged from 0 to >10 years and they reported the frequency with which they worked with this population as ‘sometimes’. One professional had experience working specifically with CTBT in a physical therapy setting 0 to 3 years prior. This user reported the frequency with which they worked with CTBT as ‘rarely’.

#### Instructor training module

Users rated the Instructor Training Module as learnable. This included the module's ability to provide users with information on the Fit ABCS programme (Mode = Excellent), how to identify appropriate programme activities (Mode = Good), physical (Mode = Excellent) and cognitive issues (Mode = Excellent) experienced by CTBT, and the role of the instructor (Mode = Good/Excellent). In addition, all users rated their general ability to work with CTBT as ‘confident’. The language used in the instructor training module was also rated as ‘very clear’ by all users. Further, the module was efficient, as indicated by ratings of the ability to navigate the module (Mode = Easy/Very Easy), and the organization and flow of its content (Mode = Excellent). Finally, users indicated their satisfaction with the instructor training module, as they most often stated that no essential (Mode = No) or desired (i.e. non-essential; Mode = No) information was missing to successfully carry out the Fit ABCS programme. User B expressed a desire for information on appropriate language to use when instructing programme activities:

*[It] may also be nice to recommend certain lingo when it comes to instruction. For example, with kids I never like when people use “weak/bad” arm versus “good/strong” arm. Maybe go over that we could make it nice by saying “make sure Mr Left or Lefty” helps out “Mr Right or Righty”.*

#### Activity library module

The quality of the visual tools (i.e. diagrams, photos and videos) and language included in the Activity Library was highly rated by users (Mode = Good/Excellent). For example, user A stated the following:

*The videos were amazing. I remember the titles of the games from my childhood but couldn't remember the rules. I found the videos very helpful and the printable PDF [of the activity description].*

While users felt that the types of visual tools included in the activity library were sufficient (i.e. is there a specific form of visual tool that you feel was missing?; Mode = No), they also indicated that some visual tools did not provide an accurate depiction of the corresponding activity (i.e. are there missing visual tools necessary for understanding of the activities?; Mode = Yes). Specifically, user C stated the following:

*If you're going to include pictures and/or videos that reflect the activity, you may want to ensure that they are accurate depictions of the activity, and easy to understand.*

Similar to the instructor training module, the activity library was rated as efficient, as indicated by ratings of the ability to navigate the library (Mode = Very Easy), and use of a filtering system to identify appropriate activities (Mode = Excellent). Users were satisfied with the activity library, as they reported that no essential (Mode = No) or desired (i.e. non-essential; Mode = No) information was missing.

#### Session planner module

The Session Planner was considered learnable and efficient, as users rated their ability to successfully use the planner as ‘comfortable’ or ‘very comfortable’ (Mode = Comfortable/Very Comfortable) and their ability to navigate the planner as ‘very easy’ (Mode = Very Easy). The Session Planner was considered satisfactory, as users indicated there were a sufficient number (Mode = Very Sufficient) and variety (Mode = Very/Completely Satisfied) of activities, and no essential (Mode = No) or desired (i.e. non-essential; Mode = No) information was missing.

#### Modifications informed by usability assessment

Two issues were identified through the usability assessment: (i) a desire for discussion of appropriate language to use when instructing programme activities and (ii) correspondence between visual tools and activities in the Activity Library Module, as the users felt some visual tools did not accurately depict the activities.

The website was modified to include examples of appropriate language to instruct programme activities. These examples promote the use of positive and encouraging language to increase participants’ physical competency and confidence. They were included in the Tips Sheet that provides easily accessible information to be used during exercise sessions. Activities included in the Activity Library Module were reviewed to identify disparate visual tools. Those visual tools that did not accurately depict their corresponding activity were replaced with more accurate iterations. Usability re-testing was not performed, as the issues identified by users were considered minor (see Methods, Usability Testing for criteria for usability re-testing).

Two changes to the Fit ABCS website proposed by professionals were not adopted due to relevancy and software constraints. These proposed changes were (i) inclusion of information on neurorehabilitation techniques and assistive devices in the Instructor Training Module and (ii) a computationally faster filtering system in the Activity Library Module. The rationale for excluding these changes is further discussed in the study limitations (pages 30–32).

## Discussion

### Main findings

Here we report on i) the state of eHealth resources to support exercise training for CTBT and ii) the development and usability of a website for community HFP to deliver exercise training to patients in community settings with the goal of improving cognitive sequelae. The scoping review revealed that there is currently a lack of eHealth resources for exercise training to improve cognitive sequelae in patients, and education and tools for professionals to deliver this training. Exercise is feasible and effective to promote cognitive improvement in CTBT, and eHealth has the potential to increase accessibility of intervention to patients in the community. Thus, there is a critical opportunity to further develop and implement eHealth resources to support accessible therapeutic exercise training for patients, the majority of which receive their ongoing rehabilitative care in the community. It is also imperative that eHealth resources be developed to provide education and tools for professionals to deliver exercise training given the demonstrated success of an instructor-led model in a hospital setting. The Fit ABCS website helps begin to meet this opportunity. The Activity Library, Session Planner and Session Reporting Modules specifically provide tools for instructors to deliver exercise training to CTBT in the community, while the Instructor Training Module provides education and training for community HFP to work with patients in exercise contexts.

The Fit ABCS website was also found to be usable, which is critical for successful implementation and feasibility.^43,57^ For web-based health interventions, it is essential to ensure users’ needs are met and that they can navigate the website efficiently and appropriately.^58^ The website was rated as clear and efficient by community HFP with expertise delivering exercise programming to cancer survivors, including the ability to navigate the website and comprehend its language and visual tools. User needs were also met, as the website was rated as learnable and satisfactory with few modifications required.

### Comparison with prior work and future directions

The development and usability of the Fit ABCS website and other eHealth resources identified in the scoping review also point towards the future possibility for exercise interventions to become accessible outside of clinical research. Indeed, home-based active video gaming and web-based coaching are feasible and effective to improve activities of daily living and motor performance among CTBT,^18,52^ and a web-based token economy increases exercise adherence in patients.^53^ The next step is to determine the feasibility of the Fit ABCS website to support HFP to deliver exercise training to CTBT in community settings. Indeed, the clinical trial of community exercise training for CTBT using the website is currently underway (see ClinicalTrials.gov: NCT05367076). Additionally, larger multi-site trials are needed and it is plausible that future directions could also include publicly or commercially available eHealth exercise training resources developed by research scientists and clinicians to promote improvements in long-term outcomes, including cognition. The use of instructor-led models to deliver public or commercially available exercise training is also plausible. Excellent examples include the Pediatric Oncology Exercise Manual^55^ and Thrive Health Services,^54^ as they successfully provide public and commercial educational resources and tools for community HFP to deliver exercise training to paediatric cancer survivors. Further, a portion of these resources and tools focuses on paediatric brain tumours and the benefits of physical activity and exercise on cognition.

### Limitations

The following limitations also merit consideration. First, the scoping review was conducted exclusively in English due to the authors’ proficiency in the language. Consequently, relevant literature in other languages may not have been included. Future research should incorporate literature available in other languages. The focus groups and usability assessment also relied on subjective data. To limit concerns about the validity and reliability of these data, the focus groups followed pre-designed semi-structured interview guides and the usability questionnaire was modelled on previous eHealth usability questionnaires with demonstrated validity and reliability.^46,47,48,49^ Additionally, while modelled on valid and reliable questionnaires, the questionnaire used in the current study was not pilot tested. Its also did not assess the confidence of HFP in their ability to work with CTBT prior to training. Including this information in the future would enable examination of potential change in HFP confidence after training. Some changes to the Fit ABCS website proposed by professionals were also not adopted due to relevancy and software constraints. Information on neurorehabilitation was not added to the Instructor Training Module because CTBT who participated in the exercise training that the Fit ABCS website supports are not on active treatment. Discussion of assistive devices was also not added because their use among these patients is rare. To date, CTBT in either trial have not used assistive devices, likely because by the time they were eligible to participate the physical difficulties they may have experienced were less severe and these devices were unnecessary. We anticipate this will also be the case for future participation in the exercise training that the website supports. Specifically, assistive devices are commonly used during in-patient rehabilitation to address physical difficulties (e.g. weakness and ataxia) that can be severe within the first year following tumour resection. During this time patients often receive adjuvant treatment, including radiation and chemotherapy, that can lead to longer than one year from surgical resection to the completion of treatment. Further, CTBT who participated in the hospital-led clinical trial from which the website content is modelled were a minimum of one year from conclusion of treatment (ClinicalTrials.gov: NCT01944761) and the current community-led feasibility trial (ClinicalTrials.gov: NCT05367076) also includes participants who are no longer on treatment. The current website technology also requires a brief wait time once each selected filter is loaded in the Activity Library Module, constraining the ability to implement a computationally faster filtering system. Nevertheless, these concerns were brought to authors’ awareness through the success of the usability assessment and will be re-considered in the future if and when updates are needed and possible. Finally, the issues identified during usability testing – one in the Tips Sheet and the other in the Activities Library Module – were considered minor and changed without re-testing by users. Given that minimal modifications were performed to the website overall, usability remains high. The usability of these minor changes will be tested in the future if and when the website is updated.

## Conclusions

There is presently no exercise intervention led by community HFP to promote improvements in long-term cognitive seqeulae in CTBT; eHealth is well positioned to support its development. The lack of community-led interventions, including but not limited to exercise, must change to improve functional outcomes and promote greater quality of life among patients. To this end, the findings of the scoping review, and the development and usability of the Fit ABCS website advance knowledge in the following ways. First, the scoping review reveals a lack of accessible eHealth resources to support exercise training as intervention for CTBT and paediatric acquired brain injury more broadly, and education and tools for HFP in the community to deliver these interventions. Second, the development and usability of the Fit ABCS website begins to address these gaps by providing a resource that is usable by HFP in the community to deliver exercise training to CTBT to improve cognitive outcomes. Most importantly, the Fit ABCS website marks one of the first of many potential eHealth resources designed to increase accessibility of therapy. CTBT stand to benefit substantially from the development of this website and similar eHealth resources, as they would afford an increase in the number of patients who have access to intervention that may improve cognition and ultimately enhance quality of life.

## Supplemental Material

sj-docx-1-dhj-10.1177_20552076241272710 - Supplemental material for A web-based resource for exercise training in children treated for brain tumours to improve cognitive sequelae: Development and usability Supplemental material, sj-docx-1-dhj-10.1177_20552076241272710 for A web-based resource for exercise training in children treated for brain tumours to improve cognitive sequelae: Development and usability by Elizabeth Cox, Marium Kiwan, Cynthia de Medeiros, Jeanne Chen-Lai, Celia Cassiani, Julie Tseng, Krista Johnston, Brian W Timmons and Jennifer N Stinson, Eric Bouffet, Donald J Mabbott in DIGITAL HEALTH

sj-docx-2-dhj-10.1177_20552076241272710 - Supplemental material for A web-based resource for exercise training in children treated for brain tumours to improve cognitive sequelae: Development and usability Supplemental material, sj-docx-2-dhj-10.1177_20552076241272710 for A web-based resource for exercise training in children treated for brain tumours to improve cognitive sequelae: Development and usability by Elizabeth Cox, Marium Kiwan, Cynthia de Medeiros, Jeanne Chen-Lai, Celia Cassiani, Julie Tseng, Krista Johnston, Brian W Timmons and Jennifer N Stinson, Eric Bouffet, Donald J Mabbott in DIGITAL HEALTH

**Figure:** Video 1.
