# Supplementary material for: A web-based resource for exercise training in children treated for brain tumours to improve cognitive sequelae: Development and usability
Source: Digit Health. 2024 Sep 5;10:20552076241272710. doi: 10.1177/20552076241272710 (PMC11387797; doi:10.1177/20552076241272710)
Supplement: sj-docx-1-dhj-10.1177_20552076241272710 - Supplemental material for A web-based resource for exercise training in children treated for brain tumours to improve cognitive sequelae: Development and usability [file sj-docx-1-dhj-10.1177_20552076241272710.docx]

**A web-based resource for exercise training in children treated for brain tumours**

**to improve cognitive sequelae: Development and usability**

**Supplemental Material**

**Supplemental Table 1.** Complete search strategies for academic literature.

| **QUERY #** | **SEARCH TERMS** | **RESULTS** |
| --- | --- | --- |
| **1** | (eHealth) OR (digital care) OR (web-based intervention) OR (digital intervention) OR (telemedicine) OR (telecare) AND (physical activity) OR (exercise) AND (acquired brain injury) | 211 |
| **2** | (eHealth) OR (digital care) OR (web-based intervention) OR (digital intervention) OR (telemedicine) OR (telecare) AND (physical activity) OR (exercise) AND (cancer) | 742 |
| **3** | (eHealth) OR (digital care) OR (web-based intervention) OR (digital intervention) OR (telemedicine) OR (telecare) AND (physical activity) OR (exercise) AND (brain tumour) | 82 |

*Note.* The academic literature search strategy reflects that performed in PsyINFO, which analogous to the database search performed in PubMed.

**Supplemental Table 2.** Content analysis of focus groups (n=3) with health and fitness professionals with experience delivering exercise programming to cancer survivors in hospital and community settings.

| **THEME** | **CATEGORY** | **EXAMPLE CODE** |
| --- | --- | --- |
| **HOSPITAL SETTING** (n=5) | |  |
| Training on the exercise intervention | Education on population (CTBT) | Physical and cognitive difficulties |
|  | Intervention goals and objectives | Increase heart rate |
|  | Session monitoring and considerations | Promote physical literary |
| Exercise session  planning | Considerations when selecting activities | Level of ability given difficulties (i.e., physical, cognitive) |
|  | Challenges to consider when selecting activities | Availability of equipment |
|  | Tools to aid in session preparation and instruction | Insights by instructors about activities  during previous sessions |
|  | Participant information | Age of participants |
| Exercise session  feedback | Information to collect | Activities that prompt high participation |
|  | Questions to ask | Which components of the session were (un)successful? |
| **COMMUNITY SETTING**^a^ (n=8, 4 per group) | |  |
| Instructor experience | Experience with children and adolescents (including CTBT) | Work context |
|  | Credentials | Professional designations |
| Instructor training | Knowledge and tools for program instruction | Boundaries (e.g., acceptable forms of physical contact) |
|  | Motivation for instructors | Benefits of the exercise for participants |
|  | Working with children | Effective communication |
| Training on the exercise intervention | Education on population (CTBT) | Physical difficulties |
|  | Intervention goals and objectives | Increase heart rate |
|  | Session monitoring and considerations | Promote physical literary |
|  | Parent/guardian support | Benefits of intervention for participant (e.g., physiological and psychological benefits of group physical exercise) |
| Venue considerations  and suitability | Intervention goals and objectives | Venue size allowing for moderate-to-vigorous exercise intensity |
|  | Venue selection | Alternative venues |
| Exercise session  planning | Participant information | Relevant medical history |
|  | Venue information | Venue size |
|  | Efficiency | Program modifications to promote participant engagement |

Abbreviation: *CTBT*=children treated for brain tumours.

^a^data from the two focus groups with professionals in community settings was combined.
